# Psychische Gesundheit von Kindern und Jugendlichen in Zeiten globaler Krisen: Ergebnisse der COPSY-Längsschnittstudie von 2020 bis 2024

**DOI:** 10.1007/s00103-025-04045-1

**Published:** 2025-04-28

**Authors:** Anne Kaman, Michael Erhart, Janine Devine, Ann-Kathrin Napp, Franziska Reiß, Steven Behn, Ulrike Ravens-Sieberer

**Affiliations:** 1 Zentrum für Psychosoziale Medizin, Klinik für Kinder- und Jugendpsychiatrie, -psychotherapie und -psychosomatik, Forschungssektion Child Public Health, Universitätsklinikum Hamburg-Eppendorf, Martinistraße 52, 20246 Hamburg, Deutschland; 2 Fachbereich Gesundheit, Erziehung und Bildung, Alice Salomon Hochschule Berlin, Berlin, Deutschland

**Keywords:** Gesundheitsbezogene Lebensqualität, Ängste, Depressive Symptome, Soziale Medien, Krisen, Health-related quality of life, Anxiety, Depressive symptoms, Social media, Crises

## Abstract

**Hintergrund:**

Die psychische Gesundheit von Kindern und Jugendlichen wird zunehmend durch globale Krisen beeinträchtigt, doch bisher gibt es nur wenige Längsschnittstudien zu diesem Thema. Ziel dieser bevölkerungsbezogenen Längsschnittstudie war es, die Entwicklung der psychischen Gesundheit in Zeiten globaler Krisen zu erforschen.

**Methoden:**

Ausgewertet wurden Daten von *n* = 2865 Familien mit Kindern und Jugendlichen im Alter von 7 bis 22 Jahren, die an mindestens einer Welle der COPSY(COrona und PSYche)-Studie von Mai 2020 (T1) bis Oktober 2024 (T7) teilgenommen haben. Im Durchschnitt nahmen die Familien an 56,7 % der Befragungswellen teil. Mithilfe von deskriptiven Statistiken und multivariaten Regressionsanalysen wurden die Veränderungen der psychischen Gesundheit sowie die Auswirkungen von krisenbedingten Sorgen und der Nutzung digitaler Medien untersucht.

**Ergebnisse:**

Die psychische Gesundheit von Kindern und Jugendlichen hat sich zu Beginn der Pandemie erheblich verschlechtert, verbesserte sich in den Folgejahren, blieb aber im Herbst 2024 im Vergleich zu den Werten vor der Pandemie weiterhin beeinträchtigt. Gleichzeitig haben Sorgen vor Kriegen, Wirtschaftskrisen und der Klimakrise zugenommen. Risikofaktoren wie eine niedrige elterliche Bildung und psychische Probleme der Eltern waren mit einer schlechteren psychischen Gesundheit assoziiert, während persönliche, familiäre und soziale Ressourcen eine schützende Wirkung hatten. Es wurde ein hoher Medienkonsum dokumentiert, der mit belastenden Erfahrungen verbunden war.

**Diskussion:**

Globale Krisen stellen eine große Herausforderung für die psychische Gesundheit von Kindern und Jugendlichen dar. Ressourcenorientierte Präventions- und Interventionsmaßnahmen sind dringend erforderlich, um sie in der Bewältigung dieser Belastungen zu unterstützen.

**Zusatzmaterial online:**

Zusätzliche Informationen sind in der Online-Version dieses Artikels (10.1007/s00103-025-04045-1) enthalten.

## Abstract (en)

**Background:**

The mental health of children and adolescents is increasingly affected by global crises, but so far there are only limited longitudinal studies on this topic. This longitudinal, population-based study aimed to explore changes in mental health during global crises.

**Methods:**

The study analyzed data from 2865 families with children and adolescents aged 7 to 22 years who participated in at least one wave of the COPSY (COvid-19 and PSYchological health) study from May 2020 (T1) to October 2024 (T7). On average, the families took part in 56.7% of the survey waves. Descriptive statistics and multivariate regression analyses were used to examine changes in mental health as well as the effects of crisis-related worries and digital media use.

**Results:**

The mental health of children and adolescents was significantly impaired at the beginning of the pandemic, improved again in the following years, but remained at an impaired level in fall 2024 compared to pre-pandemic levels. At the same time, concerns about wars, economic crises, and the climate crisis have increased significantly. Risk factors such as low parental education and parental mental health problems were associated with poorer mental health, while personal, familial, and social resources had a protective effect. A high level of media consumption was documented, which was associated with stressful experiences.

**Conclusion:**

Global crises pose significant challenges to the mental health of children and adolescents. Resource-oriented prevention and intervention efforts are urgently needed to support young people in coping with these challenges.

## Einleitung

Kinder und Jugendliche sind in der heutigen Zeit mit zahlreichen globalen Krisen konfrontiert. Die COVID-19-Pandemie hat viele junge Menschen stark geprägt und für globale Themen sensibilisiert. Die Auswirkungen auf die psychische Gesundheit waren erheblich [1–3]. Die *Lancet Psychiatry Commission on Youth Mental Health* bezeichnete dies als globale Krise der psychischen Gesundheit und forderte eine stärkere Priorisierung der Versorgung von Kindern und Jugendlichen [4]. Selbst nach Ende der Pandemie berichteten noch viele Kinder und Jugendliche von anhaltenden Belastungen [5]. Gleichzeitig fehlt Kindern und Jugendlichen die Gelegenheit zur Erholung, da sie mit neuen Krisen und Herausforderungen konfrontiert werden. Kriege in Europa und dem Nahen Osten, wirtschaftliche Unsicherheiten und die Klimakrise belasten die psychische Gesundheit von jungen Menschen zusätzlich und können zu Zukunftsängsten, Ärger, Frustration, Hoffnungslosigkeit und Traurigkeit führen. Dabei spielen auch die Beeinflussung der Wahrnehmung durch verstärkte Berichterstattung und der einfache Zugang zu Informationen über soziale Medien eine Rolle. Studien zeigen, dass ein hoher Medienkonsum mit erhöhten Angst‑, Depressions- und Stresslevels assoziiert ist [6].

Internationale Metaanalysen zeigen, dass die COVID-19-Pandemie erhebliche Herausforderungen für Kinder und Jugendliche mit sich brachte. Neben einer Abnahme der gesundheitsbezogenen Lebensqualität (gLQ) wurde ein Anstieg von Angst- und Depressionssymptomen sowie des Medienkonsums dokumentiert, insbesondere in den ersten anderthalb Jahren der Pandemie [1–3]. Die bundesweite COPSY-(COrona und PSYche‑)Studie berichtete in den Jahren 2020/2021 über eine Verschlechterung der gLQ und psychischen Gesundheit im Vergleich zu präpandemischen Daten [7]. In den Folgejahren 2022/2023 verbesserte sich die gLQ wieder und psychische Auffälligkeiten nahmen ab. Im Vergleich zu der Zeit vor der Pandemie blieb die psychische Gesundheit jedoch weiterhin beeinträchtigt [5].

Um die Auswirkungen globaler Krisen auf Kinder und Jugendliche zu erforschen, werden zunehmend internationale und nationale Studien durchgeführt. Dabei untersuchen viele dieser Studien die Folgen einzelner Krisen, etwa von Kriegen und Terror im Allgemeinen [8] oder spezifischen Konflikten wie dem Russland-Ukraine-Krieg [9]. Auch die psychischen Auswirkungen der Klimakrise [10, 11] sowie von Wirtschaftskrisen [12] stehen im Fokus dieser Forschung. Nur wenige Studien untersuchen derzeit das Zusammenwirken verschiedener globaler Krisen auf die psychische Gesundheit von Kindern und Jugendlichen. Eine Studie zeigte, dass pandemie- und klimabezogene Belastungen bei 12- bis 16-Jährigen mit erhöhten Depressions- und Angstsymptomen sowie geminderter gLQ assoziiert waren, während kriegsbezogene Belastungen vor allem Ängstlichkeit verstärkten [13]. Die *Shell Jugendstudie* zeigte Anfang 2024, dass 81 % der 12- bis 25-Jährigen Angst vor einem Krieg in Europa haben. Zudem sorgten sich 67 % um die wirtschaftliche Lage, 63 % hatten Angst vor dem Klimawandel und 64 % befürchteten eine zunehmende Feindseligkeit unter den Menschen [14]. Die Trendstudie *Jugend in Deutschland* ergab im Sommer 2024, dass 65 % der 14- bis 29-Jährigen sich Sorgen über Inflation machten, 60 % über Kriege in Europa und Nahost, 54 % über teuren/knappen Wohnraum und jeweils 49 % über die gesellschaftliche Spaltung und den Klimawandel [15].

Dennoch bleibt unklar, wie sich diese parallelen und oft miteinander verwobenen Krisen langfristig auf Kinder und Jugendliche auswirken. Auch ist wenig darüber bekannt, wie der Medienkonsum inmitten dieser sich überschneidenden Krisen die psychische Gesundheit von Kindern und Jugendlichen beeinflusst. Ziel der vorliegenden Längsschnittstudie ist es daher, einen umfassenden Einblick in die Folgen der multiplen Krisen auf die psychische Gesundheit von Kindern und Jugendlichen zu gewinnen. Im Fokus stehen folgende Fragestellungen:

1. Wie haben sich die gLQ und psychische Gesundheit von Kindern und Jugendlichen in Deutschland in Zeiten multipler globaler Krisen entwickelt? Welche alters- und geschlechtsspezifischen Unterschiede gibt es?
2. In welchem Ausmaß bereiten aktuelle Krisen (Pandemie, Kriege, wirtschaftliche Krisen, Klimakrise, Terrorismus) den Kindern und Jugendlichen Sorgen und wie haben sich diese Sorgen in den Jahren 2023 und 2024 entwickelt?
3. Wie sind krisenbezogene Zukunftsängste mit der gLQ und psychischen Gesundheit von Kindern und Jugendlichen assoziiert?
4. Welche Risikofaktoren und Ressourcen sind mit der gLQ und der psychischen Gesundheit von Kindern und Jugendlichen in Krisenzeiten assoziiert?
5. Wie sind die Nutzung und Erfahrungen in sozialen Medien mit dem Wohlbefinden von Kindern und Jugendlichen assoziiert?

## Methoden

### Studiendesign und Stichprobe

Die COPSY-Studie ist eine deutschlandweite, bevölkerungsbasierte Längsschnittstudie, die die Lebensqualität und psychische Gesundheit von Kindern und Jugendlichen untersucht. Die bisherigen 7 Erhebungen fanden zu folgenden Zeitpunkten statt: zu Beginn der COVID-19-Pandemie während eines teilweisen Lockdowns (T1: 05–06/2020), im ersten Pandemiewinter unter einem vollständigen bundesweiten Lockdown (T2: 12/2020–01/2021), im Herbst 2021 bei niedrigen Infektionszahlen und gelockerten Maßnahmen (T3: 09–10/2021), nach dem zweiten Pandemiewinter während bestehender Kontaktbeschränkungen und zu Beginn des Ukraine-Kriegs (T4: 02/2022), im Herbst 2022 während der Energiekrise und minimaler pandemiebedingter Einschränkungen (T5: 09–10/2022), im Herbst 2023 nach der offiziellen Erklärung der WHO über das Ende der Pandemie und im Kontext des eskalierten Israel-Hamas-Konflikts (T6: 10–11/2023) sowie im Herbst 2024 (T7: 10/2024). Präpandemische Vergleichsdaten wurden aus der BEfragung zum seeLischen WohLbefinden und VerhAlten (BELLA; [16]; T0: 2014–2017) herangezogen. Die Methodik der COPSY-Studie entspricht in Bezug auf die Zielpopulation sowie die Erhebungsmethoden und Instrumente der BELLA-Studie, wodurch eine Vergleichbarkeit hergestellt werden konnte.

An jeder Erhebungswelle nahmen zwischen *n* = 1586 (T1) und *n* = 1505 (T7) Familien an der Online-Befragung mittels etablierter Fragebögen teil. Bereits befragte Familien wurden zu Folgeerhebungen eingeladen, während neue Familien zur Kompensation von Drop-outs und altersbedingten Ausfällen rekrutiert wurden, um die soziodemografische Repräsentativität und Vergleichbarkeit zwischen den Befragungswellen sicherzustellen. Insgesamt beteiligten sich *n* = 2865 Familien mit Kindern und Jugendlichen im Alter von 7 bis 22 Jahren an mindestens einer Erhebungswelle (T1–T7). Neben elterlichen Angaben wurden von Jugendlichen und jungen Erwachsenen im Alter von 11 bis 22 Jahren (*n* = 1967) Selbstauskünfte erhoben. Die initiale Teilnahmequote lag bei 45,8 % und die Teilnehmenden nahmen durchschnittlich an 56,7 % der Befragungswellen (T2–T7) teil. Die Daten wurden auf Basis des Mikrozensus gewichtet, sodass die Stichprobe in den wesentlichen Merkmalen der Struktur der entsprechenden Grundgesamtheit entspricht. Weitere Details zur COPSY-Studie sind an anderer Stelle publiziert [5, 17].

### Erhebungsinstrumente

*Soziodemografische Variablen:* Es wurden das Alter und Geschlecht der Kinder und Jugendlichen sowie ihrer Eltern (jeweils im Selbstbericht) erhoben. Zusätzlich machten die Eltern Angaben zum Familienstand, Bildungsstand, Wohnraum und Migrationshintergrund.

*Psychische Gesundheit:* Die selbstberichtete gLQ von 11- bis 22-Jährigen wurde mithilfe des international anerkannten KIDSCREEN-10-Index [18] erhoben. Die Einteilung in niedrige, normale und hohe gLQ erfolgte anhand von Referenzdaten der BELLA-Studie (normale gLQ = M_BELLA_ ± 1SD_BELLA_). Psychische Auffälligkeiten von 7‑ bis 22-Jährigen wurden durch die deutsche Version des elternberichteten Strengths and Difficulties Questionnaire (SDQ; [19]) erfasst, der einen Gesamtscore basierend auf 20 Items umfasst. Höhere Werte deuten auf stärkere Auffälligkeiten hin. Unter Verwendung etablierter Cut-offs [20] wurden die Teilnehmenden in 2 Gruppen *mit* und *ohne* psychische Auffälligkeiten eingeteilt (13–40 Pkt. = *auffällig/grenzwertig* vs. 0–12 Pkt. = *unauffällig*). Angstsymptome wurden im Selbstbericht mittels der Subskala zu generalisierter Ängstlichkeit der deutschen Version des Screen for Child Anxiety Related Disorders (SCARED; [21]) erfasst. Gruppen von Kindern *mit* (9–18 Pkt.) und *ohne* (0–8 Pkt.) generalisierte Ängstlichkeit wurden basierend auf etablierten Cut-offs definiert [21]. Depressive Symptome wurden mit der deutschen Version der Center for Epidemiological Studies Depression Scale (CES–DC; [22]) im Selbstbericht erfasst. Teilnehmende wurden in 2 Gruppen *mit* (15–28 Pkt.) und *ohne* (1–14 Pkt.) depressive Symptome eingeteilt [23]. Über ein Item des KIDSCREEN-10-Index [18] wurde erhoben, ob die Kinder und Jugendlichen sich in der letzten Woche einsam gefühlt haben.

*Krisenbezogene Zukunftsängste und Sorgen:* Die Sorgen der Kinder und Jugendlichen in Bezug auf spezifische Krisen (z. B. Pandemie, Kriege, Wirtschaftskrise, Klimakrise, Terrorismus) wurden mit eigens für die Studie neu entwickelten Einzelitems erfasst. Die Intensität der Sorgen hinsichtlich jeder spezifischen Krise gaben die Teilnehmenden auf einer 5‑stufigen Skala von *überhaupt nicht* bis *sehr *an. Zusätzlich wurden krisenbezogene Zukunftsängste mithilfe einer adaptierten Version der Dark-Future Scale für Kinder (DFS‑K; [24, 25]) erhoben. Diese umfasst 4 Items zur Dauer der Krisen, einer allgemeinen Verschlechterung des Lebens sowie zu finanziellen und persönlichen Schwierigkeiten, die angesichts der aktuellen Krisen in der Zukunft auftreten können. Die Zustimmung zu diesen Aussagen wurde von den Befragten auf einer 4‑stufigen Skala von *stimmt nicht* bis *stimmt genau* erfragt.

*Risikofaktoren:* Es wurden Angaben zum Bildungsstand, Migrationshintergrund und Wohnraum der Eltern erfasst. Der elterliche Bildungsstand wurde basierend auf der CASMIN Klassifikation (Comparative Analyses of Social Mobility in Industrial Nations; [26]) erfasst. Diese unterscheidet 9 Bildungsklassen basierend auf der schulischen und beruflichen Qualifikation. Anhand der Angaben der Eltern erfolgte damit eine Klassifikation in 3 Gruppen (geringer, mittlerer und hoher Bildungsstand). Kinder und Jugendliche, die in Haushalten mit niedrigem Bildungsstand leben und entweder einen Migrationshintergrund haben oder über weniger als 20 m^2^ Wohnraum pro Haushaltsmitglied verfügen, wurden als Risikogruppe eingestuft. Zudem wurden elterliche depressive Symptome mit dem Patient Health Questionnaire (PHQ‑8; [27]) erhoben. Basierend auf 8 Items wurde ein Summenwert mit Werten zwischen 0 und 24 berechnet. Kinder und Jugendliche, deren Eltern von depressiven Symptomen, einer psychischen Erkrankung oder starken Sorgen aufgrund aktueller Krisen berichten, gehörten ebenfalls zur Risikogruppe.

*Ressourcen:* Personale Ressourcen wie Problemlösungsfähigkeiten und Optimismus der Kinder und Jugendlichen wurden mit der Personale Ressourcen Skala [28] erfasst. Die wahrgenommene soziale Unterstützung wurde mit 4 Items der deutschsprachigen Version der Social Support Scale [29] gemessen. Zur Beurteilung des Familienklimas wurden 4 Items aus der Subskala *Familiärer Zusammenhalt* der Familienklimaskala verwendet [30]. Für alle 3 Skalen wurden Summenwerte berechnet, wobei höhere Werte auf stärker ausgeprägte Ressourcen wie Problemlösefähigkeiten, Optimismus, soziale Unterstützung und Familienzusammenhalt hinweisen. Als Gruppe mit ausgeprägten Ressourcen wurden die Kinder und Jugendlichen klassifiziert, die in jeder der 3 Skalen höhere Werte angaben, als es den entsprechenden Durchschnitten in der präpandemischen BELLA-Studie entspricht (personale Ressourcen > 58 Pkt.; soziale Unterstützung > 75 Pkt.; Familienklima > 48 Pkt.).

*Mediennutzung:* Die Kinder und Jugendlichen wurden mittels selbst entwickelter Items gefragt, wie viele Stunden am Tag sie digitale Medien für (i) private Angelegenheiten und (ii) schulische Zwecke nutzen. Darüber hinaus wurden die Belastungen durch Inhalte in den sozialen Medien sowie durch Ausgrenzung und Abwertung mittels eigenentwickelter Items erfasst.

### Statistische Analysen

Die Veränderung der gLQ und psychischen Gesundheit wurde anhand der prozentualen Verteilung auffälliger Werte (geminderte gLQ, psychische Auffälligkeiten, ängstliche und depressive Symptome, Einsamkeit) über alle Erhebungszeitpunkte (T1–T7) analysiert und mit präpandemischen Daten aus der BELLA-Studie verglichen. Unterschiede wurden mittels Chi-Quadrat-Tests geprüft und Effektstärken durch Cramers V berechnet. Alters- und geschlechtsspezifische Unterschiede wurden in stratifizierten Analysen untersucht. Itemantworten zu Sorgen um spezifische Krisen im Herbst 2024 (T7) wurden zunächst deskriptiv analysiert und anschließend mit den diesbezüglichen Itemantworten aus Herbst 2023 (T6) verglichen. Unterschiede zwischen T6 und T7 wurden mittels Chi-Quadrat-Tests geprüft und Effektstärken durch Cramers V berechnet. Basierend auf den Itemantworten aus Herbst 2024 (T7) wurde der Zusammenhang zwischen krisenbezogenen Zukunftsängsten (erfasst mittels der DFS-K) und psychischer Gesundheit (erfasst durch den KIDSCREEN, SDQ, SCARED und CES-DC) durch logistische Regressionsanalysen untersucht, wobei Alter und Geschlecht kontrolliert wurden. Effektgrößen wurden mittels Cohens d berechnet. In multiplen logistischen Regressionsanalysen wurde untersucht, ob die Zugehörigkeit zur Risikogruppe mit einer erhöhten oder verringerten „Chance“ (OR) für eine geminderte gLQ, psychische Auffälligkeiten sowie ängstliche und depressive Symptome assoziiert ist. Alter, Geschlecht und deren Interaktion wurden als Kovariaten in die Analysen einbezogen. In einem erweiterten Modell wurde zusätzlich das Vorhandensein ausreichender personaler, familiärer und sozialer Ressourcen als weiterer Prädiktor in die Regressionsmodelle integriert. Die Itemantworten zur Nutzung digitaler Medien und zu Erfahrungen in sozialen Medien wurden deskriptiv ausgewertet.

## Ergebnisse

### Soziodemografische Merkmale

Insgesamt nahmen *n* = 2865 Familien mit Kindern und Jugendlichen im Alter von 7 bis 22 Jahren (*M* = 14,4; *SD* = 4,6; 49,7 % weiblich) an mindestens einer der 7 Befragungswellen (T1–T7) der COPSY-Studie teil. Von diesen beantworteten *n* = 1967 Jugendliche und junge Erwachsene im Alter von 11 bis 22 Jahren (*M* = 16,8; *SD* = 3,2; 50,9 % weiblich) ihre Fragebögen selbst. Die Mehrheit der Eltern hatte einen mittleren Bildungsstand. Etwa ein Fünftel der Kinder und Jugendlichen hatte einen Migrationshintergrund und ein Fünftel der Eltern war alleinerziehend. Weitere soziodemografische Angaben sind in Tab. 1 dargestellt.

**Tab. 1:** Soziodemografische Charakteristika der Stichprobe der COPSY-Studie (T1–T7)

	Kinder und Jugendliche (Elternbefragung; *n* = 2865)		Kinder, Jugendliche und junge Erwachsene (Selbstbefragung; *n* = 1967)	
	*n* (%)	*M* (SD)	*n* (%)	*M* (SD)
*Alter*	–	14,4 (4,6)	–	16,8 (3,2)
7–10 Jahre	693 (24,2)	–	–	–
11–13 Jahre	613 (21,4)	–	424 (21,6)	–
14–17 Jahre	612 (21,4)	–	596 (30,3)	–
18–22 Jahre	947 (33,1)	–	947 (48,1)	–
*Geschlecht*				
Männlich	1425 (49,7)	–	951 (48,3)	–
Weiblich	1424 (49,7)	–	1001 (50,9)	–
Divers	16 (0,6)	–	15 (0,8)	–
Alter der Eltern	–	45,4 (7,3)	–	49,0 (7,3)
*Geschlecht der Eltern*				
Männlich	1284 (44,8)	–	862 (44,0)	–
Weiblich	1578 (55,1)	–	1103 (55,9)	–
Divers	3 (0,1)	–	2 (0,1)	–
*Bildung der Eltern*				
Gering	430 (15,1)	–	329 (16,8)	–
Mittel	1698 (59,6)	–	1154 (58,8)	–
Hoch	719 (25,3)	–	479 (24,4)	–
*Migrationshintergrund*				
Nein	2348 (82,5)	–	1613 (82,8)	–
Ja	498 (17,5)	–	336 (17,2)	–
*Alleinerziehend*				
Nein	2369 (82,7)	–	1584 (80,5)	–
Ja	496 (17,3)	–	383 (19,5)	–

*M* Mittelwert, *SD* Standardabweichung

### Veränderungen der gesundheitsbezogenen Lebensqualität und der psychischen Gesundheit

**Abb. 1:**
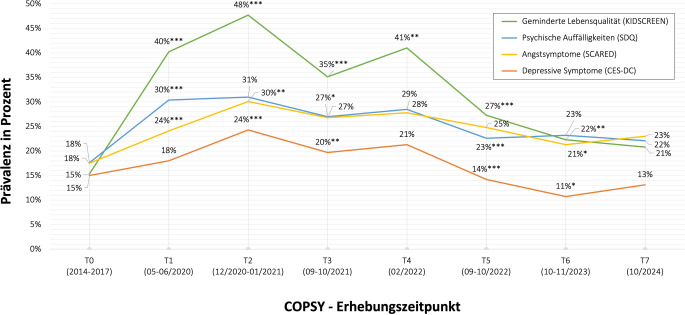
Längsschnittliche Verlaufsergebnisse des Anteils der Kinder und Jugendlichen mit einer geminderten gesundheitsbezogenen Lebensqualität, psychischen Auffälligkeiten, Angstsymptomen und depressiven Symptomen. Ergebnisse der BELLA-Studie (2014–2017) und der COPSY-Studie (2020–2024). *Anmerkung:* Statistisch signifikante Veränderungen wurden zwischen T0 und T1 gefunden für gLQ (*p* < 0,001, V = 0,28), psychische Auffälligkeiten (*p* < 0,001, V = 0,15), ängstliche (*p* < 0,001, V = 0,08) und depressive Symptome (*p* = 0,075, V = 0,04), von T1 zu T2 für gLQ (*p* < 0,001, V = 0,08), ängstliche (*p* = 0,002, V = 0,08) und depressive Symptome (*p* < 0,001, V = 0,08), von T2 zu T3 für gLQ (*p* < 0,001, V = 0,13), psychische Auffälligkeiten (*p* = 0,013, V = 0,04) und depressive Symptome (*p* = 0,007, V = 0,06), von T3 zu T4 für gLQ (*p* = 0,004, V = 0,06), von T4 zu T5 für gLQ (*p* < 0,001, V = 0,14), psychische Auffälligkeiten (*p* < 0,001, V = 0,07) und depressive Symptome (*p* < 0,001, V = 0,09) und von T5 zu T6 für gLQ (*p* = 0,005, V = 0,06), ängstliche (*p* = 0,047, V = 0,04) und depressive Symptome (*p* = 0,010, V = 0,05). Von T6 zu T7 gab es keine statistisch signifikanten Veränderungen. * *p* < 0,05; ** *p* < 0,01; *** *p* < 0,001. (Eigene Abbildung)

Die gLQ (gemessen mit dem KIDSCREEN) und die psychische Gesundheit (gemessen mit dem SDQ, SCARED und CES-DC) der Kinder und Jugendlichen verschlechterten sich zu Beginn der Pandemie im Vergleich zu präpandemischen Daten (2014–2017) der BELLA-Studie deutlich. Abb. 1 zeigt die relativen Anteile derjenigen Kinder und Jugendlichen, welche gemäß der im Methodenteil beschriebenen Cut-offs eine geminderte Lebensqualität, psychische Auffälligkeiten sowie erhöhte ängstliche bzw. depressive Symptome aufweisen. Die höchsten Prävalenzen wurden im Winter 2020/2021 (T2) festgestellt: Fast die Hälfte (48 %) berichtete von einer niedrigen gLQ, jeweils etwa ein Drittel gab psychische Auffälligkeiten (31 %) und Ängste (30 %) und ein Viertel (24 %) depressive Symptome an.

In den Jahren 2022 und 2023 verbesserten sich die gLQ und die psychische Gesundheit der Kinder und Jugendlichen. Dieser Trend der Verbesserung setzte sich im letzten Jahr bis Herbst 2024 (T7) nicht weiter fort. 21 % der Kinder und Jugendlichen gaben weiterhin eine geminderte gLQ an, 22 % gaben psychische Auffälligkeiten und 23 % Angstsymptome an. Damit liegen die Prävalenzen weiterhin etwa 5 % über den präpandemischen Werten der BELLA-Studie. Lediglich für depressive Symptome zeigte sich eine Verbesserung gegenüber dem präpandemischen Niveau, wenngleich im Herbst 2024 (T7) wieder ein ansteigender (nicht signifikanter) Trend zu beobachten ist (Abb. 1).

Darüber hinaus berichteten im Herbst 2024 (T7) 21 % der Kinder und Jugendlichen, sich manchmal, oft oder immer einsam zu fühlen. Damit liegt die Prävalenz von Einsamkeit zwar unter den Werten zu Beginn der Pandemie (T1: 35 %, T2: 39 %), bleibt jedoch weiterhin höher als vor der Pandemie (14 %).

### Alters- und geschlechtsspezifische Unterschiede in der gesundheitsbezogenen Lebensqualität und der psychischen Gesundheit

Jugendliche im Alter von 14 bis 17 Jahren hatten über den Zeitverlauf insgesamt häufiger eine geminderte gLQ als Kinder im Alter von 11 bis 13 Jahren (s. Onlinematerial Abb. S1). Psychische Auffälligkeiten waren vor allem bei jüngeren Kindern (7–10 Jahre und 11–13 Jahre) deutlich häufiger (s. Onlinematerial Abb. S2). Hinsichtlich der Angstsymptome gab es keine klaren altersbedingten Unterschiede (s. Onlinematerial Abb. S3). Depressive Symptome traten am häufigsten bei Jugendlichen im Alter von 14 bis 17 Jahren sowie bei jungen Erwachsenen auf (s. Onlinematerial Abb. S4). Die geschlechtsspezifische Analyse zeigte, dass Mädchen insgesamt stärker beeinträchtigt waren, häufiger eine geminderte gLQ sowie höhere Raten an depressiven Symptomen und Ängsten hatten (s. Onlinematerial Abb. S5). Die Signifikanzangaben aller genannten Unterschiede finden sich in den Abbildungsbeschreibungen im Onlinematerial.

### Sorgen aufgrund aktueller Krisen (Herbst 2023 vs. Herbst 2024)

**Abb. 2:**
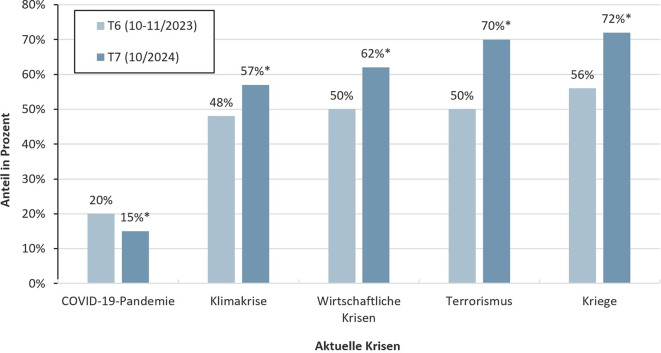
Anteil der Kinder und Jugendlichen, die aufgrund aktueller Krisen angeben, mittelmäßig bis sehr besorgt zu sein. Ergebnisse der COPSY-Studie (2023–2024). *Anmerkung:* * signifikanter Unterschied von COPSY-Erhebungszeitpunkt T7 im Vergleich zu T6 mit einem Signifikanzniveau von *p* < 0,001. (Eigene Abbildung)

Im Herbst 2023 (T6) gab jeweils etwa die Hälfte (48–56 %) der Kinder und Jugendlichen an, dass sie aufgrund von Kriegen, Terrorismus, wirtschaftlichen Krisen und der Klimakrise *mittelmäßig, ziemlich* oder *sehr* besorgt seien (Abb. 2). Diese Sorgen haben im Herbst 2024 (T7) signifikant zugenommen (*p* < 0,001; V = 0,11–0,28). Die Effektstärken lagen im schwachen bis moderaten Bereich. So äußerten 72 % der Kinder und Jugendlichen Sorgen in Bezug auf Kriege, gefolgt von Sorgen im Zusammenhang mit Terrorismus (70 %), wirtschaftlichen Krisen (62 %) und der Klimakrise (57 %). Sorgen aufgrund der COVID-19-Pandemie haben hingegen signifikant abgenommen (*p* < 0,001; V = 0,06) und wurden nur noch von 15 % der Kinder und Jugendlichen als *mittelmäßig, ziemlich* oder *sehr* besorgniserregend berichtet.

### Krisenbezogene Zukunftsängste und psychische Gesundheit

Kinder und Jugendliche, die im Herbst 2024 (T7) unter krisenbezogenen Zukunftsängsten (gemessen mit der DFS-K) litten, zeigten häufiger eine geminderte gLQ, psychische Auffälligkeiten sowie Ängste und depressive Symptome. So ergab die logistische Regressionsanalyse, dass ein Anstieg des Durchschnittswertes der krisenbezogenen Zukunftsängste um 1 mit einer 1,8fach höheren Wahrscheinlichkeit für eine geminderte gLQ assoziiert war. Etwas höhere Zusammenhänge fanden sich für psychische Auffälligkeiten (OR = 2,0), depressive Symptome (OR = 2,4) und Angst (OR = 3,0; Tab. 2). Trotz der niedrigen (Pseudo‑)Varianzaufklärung der logistischen Regressionsmodelle von 0,06 bis 0,16 können die OR als mittlere Effekte betrachtet werden [31].

**Tab. 2:** Assoziationen zwischen krisenbezogenen Zukunftsängsten und psychischer Gesundheit von Kindern und Jugendlichen im Herbst 2024. Ergebnisse der COPSY-Studie (2024)

	gLQ (KIDSCREEN-10)		Psychische Auffälligkeiten (SDQ)		Depressive Symptome (CES-DC)		Angstsymptome (SCARED)	
	OR	95 % KI	OR	95 % KI	OR	95 % KI	OR	95 % KI
Geschlecht								
Männlich (Ref.)	–	–	–	–	–	–	–	–
Weiblich	1,457	*1,07–1,98*	1,170	0,81–1,68	1,719	*1,18–2,51*	1,896	*1,39–2,58*
Altersgruppen								
11–13 Jahre (Ref.)	–	–	–	–	–	–	–	–
14–17 Jahre	1,085	0,74–1,60	0,834	0,54–1,29	1,289	0,79–2,10	0,794	0,55–1,16
18–22 Jahre	1,256	0,84–1,87	*0,590*	*0,36–0,96*	1,480	0,90–2,44	*0,624*	*0,49–1,09*
Krisenbezogene Zukunftsängste	*1,809*	*1,45–2,26*	1,997	*1,54–2,60*	2,443	*1,85–3,22*	3,001	*2,37–3,80*
Hosmer-Lemeshow GoF	–	*p* = 0,648	–	*p* = 0,469	–	*p* = 0,067	–	*p* = 0,197
Nagelkerke R‑Square	–	0,058	–	0,056	–	0,102	–	0,159

Die kursiv dargestellten Zahlen zeigen statistisch signifikante Ergebnisse an

*Anmerkung:* multiple logistische Regression zu Assoziationen zwischen krisenbezogenen Zukunftsängsten und psychischer Gesundheit von Kindern und Jugendlichen, kontrolliert für Alters- und Geschlechtsgruppen

*gLQ* gesundheitsbezogene Lebensqualität; *OR* Odds Ratio; *KI* Konfidenzintervall;* GoF* Goodness of Fit

### Risikofaktoren und Ressourcen für die psychische Gesundheit

Etwa 17 % der Kinder und Jugendlichen waren im Herbst 2024 (T7) im Hinblick auf ihre psychische Gesundheit besonders vulnerabel und gehörten zu einer Risikogruppe. Risikofaktoren waren eine geringe Bildung der Eltern, psychische Belastung der Eltern, ein Migrationshintergrund und ein beengter Wohnraum. Diese Kinder und Jugendlichen hatten ein 1,9- bis 2,7fach erhöhtes Risiko (OR) für eine geminderte gLQ (OR = 1,9), psychische Auffälligkeiten (OR = 2,7) sowie ängstliche (OR = 2,2) und depressive Symptome (OR = 2,5). Hingegen hatten Kinder und Jugendliche mit ausgeprägten personalen, familiären und sozialen Ressourcen ein 5‑ bis 10fach geringeres Risiko für eine geminderte gLQ (OR = 0,1), psychische Auffälligkeiten (OR = 0,1), ängstliche (OR = 0,2) und depressive Symptome (OR = 0,1).

### Nutzung digitaler Medien und Erfahrungen in sozialen Medien

**Abb. 3:**
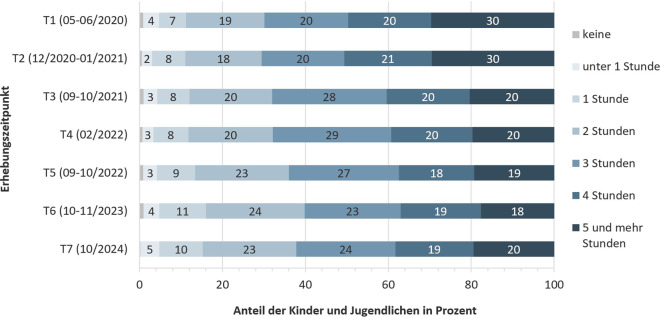
Längsschnittlicher Verlauf der selbstberichteten Nutzung digitaler Medien für private Angelegenheiten von Kindern und Jugendlichen. Ergebnisse der COPSY-Studie (2020–2024). (Eigene Abbildung)

**Abb. 4:**
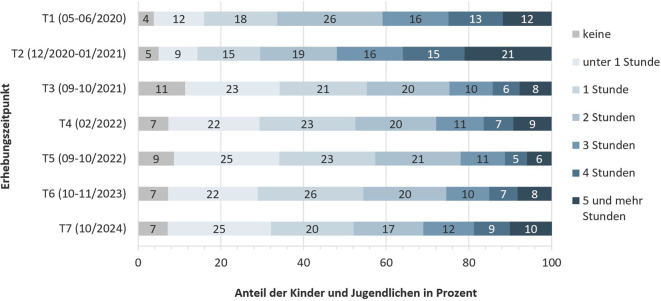
Längsschnittlicher Verlauf der selbstberichteten Nutzung digitaler Medien für schulische Zwecke von Kindern und Jugendlichen. Ergebnisse der COPSY-Studie (2020–2024). (Eigene Abbildung)

Abb. 3 und 4 zeigen die selbstberichteten Angaben der Kinder und Jugendlichen zu ihrem digitalen Mediennutzungsverhalten. Nahezu 40 % der Kinder und Jugendlichen gaben an, digitale Medien im Herbst 2024 (T7) mindestens 4 h am Tag für private Angelegenheiten zu nutzen (Abb. 3). Davon nutzte ein Fünftel der Kinder und Jugendlichen digitale Medien sogar mindestens 5 h am Tag. Im Vergleich zu den vorherigen Jahren scheint der Medienkonsum weitestgehend konstant geblieben zu sein (mit etwas höheren Nutzungszeiten zu Beginn der Pandemie). Darüber hinaus gab der Großteil der Kinder und Jugendlichen an, digitale Medien zusätzlich 1–2 h am Tag für schulische Zwecke zu nutzen (Abb. 4). Im Herbst 2024 (T7) gaben 32 % der Kinder und Jugendlichen an, dass ihnen in den sozialen Medien oft Inhalte begegneten, die sie belasten würden. Weiterhin gaben 21 % der Kinder und Jugendlichen an, dass sie sich belastet fühlen würden, weil sie in sozialen Medien Ausgrenzung und Abwertung erfahren. Fast ein Viertel (23 %) gab an, dass ihnen die Nutzung sozialer Medien nicht gut täte.

## Diskussion

Die vorliegende Studie hatte das Ziel, die psychosozialen Folgen multipler globaler Krisen auf Kinder und Jugendliche in Deutschland umfassend zu analysieren. Die Ergebnisse zeigen, dass die gLQ und die psychische Gesundheit der Kinder und Jugendlichen zu Beginn der Pandemie deutlich beeinträchtigt waren, sich in den Folgejahren zunächst wieder verbessert haben, im Herbst 2024 jedoch weiterhin auf einem im Vergleich zu präpandemischen Werten erhöhten Niveau verblieben. Besonders betroffen waren dabei Kinder und Jugendliche mit ausgeprägten Zukunftsängsten. Gleichzeitig haben Sorgen aufgrund von Kriegen, Terrorismus, wirtschaftlichen Krisen und der Klimakrise deutlich zugenommen. Risikofaktoren wie eine geringe elterliche Bildung und psychische Belastung der Eltern waren mit einer schlechteren psychischen Gesundheit assoziiert, während personale, familiäre und soziale Ressourcen schützend wirkten und mit einem geringeren Risiko für psychische Belastungen assoziiert waren. Gleichzeitig wurde ein hoher Medienkonsum dokumentiert, der bei einem Teil der Kinder und Jugendlichen mit belastenden Erfahrungen einhergeht.

Die Daten der COPSY-Studie zeigen, dass sich die gLQ und psychische Gesundheit von Kindern und Jugendlichen während der Pandemie 2020/2021 im Vergleich zu der Zeit davor deutlich verschlechterten. Dies zeigt sich in einer bis zu 3fach erhöhten Anzahl an Befragten mit Beeinträchtigungen sowie in statistisch signifikanten Effektstärken von mittlerer Größe. Zwar verbesserte sich die psychische Gesundheit 2022/2023 wieder, doch seit 2023 bleibt etwa ein Fünftel der Kinder und Jugendlichen weiterhin psychisch beeinträchtigt. Diese Ergebnisse decken sich mit den Befunden des *Deutschen Schulbarometers*, das bei 21 % der 8‑ bis 17-Jährigen Hinweise auf psychische Auffälligkeiten feststellte [32].

Ein weiterer positiver Trend zeichnet sich im Herbst 2024 in der COPSY-Studie leider nicht ab. Die Prävalenzen für eine geminderte gLQ sowie psychische Auffälligkeiten und Ängste lagen weiterhin etwa 5 % über den präpandemischen Werten. Ein Grund dafür könnte sein, dass Kinder und Jugendliche zunehmend mit neuen globalen Krisen konfrontiert werden und ein Teil von ihnen bislang weder die Zeit noch die Bedingungen hatte, sich psychisch zu erholen. Besonders alarmierend ist, dass laut *Deutschem Schulbarometer* 43 % der Lehrkräfte angeben, es fehle an ausreichenden Unterstützungsangeboten durch Schulpsycholog:innen und -sozialarbeiter:innen [32]. Gleichzeitig müssen betroffene Kinder oft bis zu 5 Monate auf einen Therapieplatz warten. Hier besteht dringender Handlungsbedarf. Die *Lancet Psychiatry Commission on Youth Mental Health *ruft eindringlich dazu auf, die psychische Gesundheit von Kindern und Jugendlichen zu einer prioritären gesellschaftlichen Aufgabe zu machen [4].

Die alters- und geschlechtsstratifizierten Analysen zeigen darüber hinaus, dass jüngere Kinder häufiger allgemeine psychische Auffälligkeiten aufweisen, während Jugendliche häufiger eine geminderte Lebensqualität und depressive Symptome berichteten. Zudem waren Mädchen insgesamt stärker psychisch belastet als Jungen. Diese Alters- und Geschlechtsunterschiede sollten bei der Entwicklung gezielter Präventions- und Interventionsprogramme zur Förderung der psychischen Gesundheit in Bildungs‑, Freizeit- und Gesundheitseinrichtungen berücksichtigt werden.

Des Weiteren zeigen die Ergebnisse der vorliegenden Studie – im Einklang mit der Trendstudie *Jugend in Deutschland* [15] und der *Shell Jugendstudie* [14] –, dass sich viele Kinder und Jugendliche um die aktuellen globalen Krisen sorgen. Im Vergleich zur letzten Erhebung der COPSY-Studie im Herbst 2023 (T6) nahmen die Sorgen im Herbst 2024 (T7) signifikant zu. Eine ähnliche Zunahme der Sorgen zeigt auch die Trendstudie *Jugend in Deutschland* [15]. Diese Entwicklung sollte von den Verantwortlichen in unserer Gesellschaft ernst genommen werden. Insbesondere da die vorliegende Studie auch zeigt, dass krisenbezogene Zukunftsängste mit einem etwa 2‑ bis 3fach erhöhten Risiko für eine geminderte gLQ, psychische Auffälligkeiten sowie ängstliche und depressive Symptome assoziiert sind. Dieses Ergebnis stimmt mit den Befunden von Lass-Hennemann et al. [13] überein, die Assoziationen zwischen pandemie- und klimabezogenem Stress und einer schlechteren gLQ sowie erhöhter Ängstlichkeit und Depressivität fanden. Diese Ergebnisse unterstreichen einmal mehr, warum die heutige Generation der Kinder und Jugendlichen in Krisenzeiten dringend Unterstützung braucht.

Die Ergebnisse der vorliegenden Studie zeigen jedoch auch positive Entwicklungen: Obwohl die Prävalenzen für eine geminderte gLQ und allgemeine psychische Auffälligkeiten sowie Ängste noch immer höher liegen als vor der Pandemie, zeigten sich bei depressiven Symptomen seit 2023 – nach der offiziellen Erklärung über das Ende der Pandemie durch die Weltgesundheitsorganisation (WHO) – erste Anzeichen einer Erholung. Dies könnte darauf hindeuten, dass sich Kinder und Jugendliche nach einem kritischen Lebensereignis an neue Umstände anpassen, resilient werden oder sich daran gewöhnen [33, 34]. Darüber hinaus deuten die aktuellen Ergebnisse darauf hin, dass Kinder und Jugendliche mit hohen personalen, familiären und sozialen Ressourcen eine signifikant bessere psychische Gesundheit haben. Besonders zuversichtliche junge Menschen, die viel Zeit mit der Familie verbringen und sich gut unterstützt fühlen, haben ein 5‑ bis 10fach geringeres Risiko für eine geminderte gLQ, psychische Auffälligkeiten sowie ängstliche und depressive Symptome. Die Wirksamkeit dieser Ressourcen scheint in den aktuellen globalen Krisenzeiten besonders stark ausgeprägt zu sein. Dies spricht dafür, dass Familien und soziale Kontexte (z. B. Bildungs- und Freizeiteinrichtungen) durch die Förderung von Selbstwirksamkeit und Unterstützung in Krisenzeiten einen wesentlichen positiven Einfluss auf die psychische Gesundheit von Kindern und Jugendlichen haben können.

Gleichzeitig zeigt die vorliegende Studie jedoch auch, dass etwa 17 % der Kinder und Jugendlichen besonders vulnerabel in Bezug auf ihre psychische Gesundheit sind und somit zu einer Risikogruppe gehören. Besonders betroffen sind Kinder und Jugendliche, deren Eltern eine geringe Bildung haben, psychisch belastet sind, einen Migrationshintergrund aufweisen oder in beengten Wohnverhältnissen leben. Diese Gruppe weist ein 1,9- bis 2,7fach erhöhtes Risiko für eine geminderte gLQ, psychische Auffälligkeiten sowie ängstliche und depressive Symptome auf. Es ist daher wichtig, diese vulnerablen Kinder und Jugendlichen in Freizeit‑, Bildungs- und Gesundheitseinrichtungen besonders zu unterstützen, indem gezielt zusätzliche Ressourcen bereitgestellt werden, um ihnen die gleiche Chance auf gute psychische Gesundheit zu bieten wie ihren ressourcenstärkeren Gleichaltrigen.

Zuletzt zeigt die vorliegende Studie, dass im Herbst 2024 rund 40 % der Kinder und Jugendlichen täglich mehr als 4 h digitale Medien nutzen und etwa 20 % sogar mehr als 5 h. Dies deckt sich mit den Befunden der *JIM-*Studie aus dem Jahr 2023, wonach Jugendliche in ihrer Freizeit täglich durchschnittlich 224 min online verbringen [35]. Dieses Mediennutzungsverhalten überschreitet deutlich die nationalen Empfehlungen des Bundesinstituts für öffentliche Gesundheit (BIÖG, ehemals BZgA; [36]) sowie der Ärzteschaft [37]. Zahlreiche Studien haben einen negativen Zusammenhang zwischen hohem Medienkonsum und psychischer Gesundheit gezeigt, etwa in Bezug auf vermehrte Ängste und depressive Symptome [38, 39]. Die COPSY-Studie bestätigt, dass etwa ein Drittel der Kinder und Jugendlichen sich durch die Inhalte, die sie in sozialen Medien sehen, belastet fühlt. Wie genau Kinder und Jugendliche durch die mediale Berichterstattung von globalen Krisen belastet werden, sollte dringend weiter erforscht werden.

Zu den Stärken der vorliegenden Studie gehören das longitudinale Design der COPSY-Studie, die große bevölkerungsbasierte Stichprobe, die Verwendung etablierter und validierter Instrumente und der lange Erhebungszeitraum, der sowohl die Pandemie als auch nachfolgende globale Krisen umfasst. Darüber hinaus ermöglicht die Verfügbarkeit von präpandemischen Daten aus der BELLA-Studie aussagekräftige Vergleiche. Die Studie weist jedoch auch Einschränkungen auf: So können keine kausalen Zusammenhänge zwischen den sich gegenseitig verstärkenden Krisen und der psychischen Gesundheit von Kindern und Jugendlichen hergestellt werden und die Ergebnisse sind möglicherweise nicht auf andere Länder übertragbar. Darüber hinaus können die ermittelten Zusammenhänge und Unterschiede, gemäß den Effektstärkemaßen, nur als kleine bis mittlere Effekte interpretiert werden.

## Fazit

Zusammenfassend verdeutlichen die Ergebnisse der vorliegenden Studie, dass auch im Herbst 2024 ein Fünftel der Kinder und Jugendlichen in Deutschland weiterhin psychisch belastet ist. Besonders betroffen sind sozial benachteiligte Kinder sowie Kinder psychisch belasteter Eltern. Viele von ihnen äußern Sorgen über aktuelle globale Krisen, insbesondere Kriege, den Klimawandel und wirtschaftliche Unsicherheiten.

Die Ergebnisse unterstreichen die dringende Notwendigkeit konkreter Maßnahmen zur Förderung der psychischen Gesundheit junger Menschen. Politik, Gesellschaft und Bildungseinrichtungen sollten gezielt evidenzbasierte Präventionsprogramme ausbauen. Dabei sind insbesondere folgende Maßnahmen essenziell:

1. Stärkung personaler und sozialer Ressourcen durch Programme zur Förderung von Selbstwirksamkeit und Resilienz;
2. Ausbau niedrigschwelliger Unterstützungsangebote, wie Schulpsychologie, psychosoziale Beratungsstellen und Online-Hilfsangebote;
3. Förderung der Medienkompetenz, um Kinder und Jugendliche im Umgang mit sozialen Medien und krisenbezogenen Informationen zu stärken.

Die Ergebnisse der COPSY-Studie sind ein dringender Appell an Politik und Gesellschaft, die psychische Gesundheit von Kindern und Jugendlichen zu priorisieren – nicht nur für ihre individuelle Zukunft, sondern auch für die Stabilität und das Wohlergehen der Gesellschaft.

## Supplementary Information

Zusätzliches Onlinematerial: Psychische Gesundheit von Kindern und Jugendlichen in Zeiten globaler Krisen: Ergebnisse der COPSY-Längsschnittstudie von 2020 bis 2024
