# Supplementary material for: Mental health of children and adolescents in times of global crises: findings from the longitudinal COPSY study from 2020 to 2024
Source: Bundesgesundheitsblatt Gesundheitsforschung Gesundheitsschutz. 2025 Apr 28;68(6):670–80. [Article in German] doi: 10.1007/s00103-025-04045-1 (PMC12129870; doi:10.1007/s00103-025-04045-1)
Supplement: Supplementary file 1 — Zusätzliches Onlinematerial: Psychische Gesundheit von Kindern und Jugendlichen in Zeiten globaler Krisen: Ergebnisse der COPSY-Längsschnittstudie von 2020 bis 2024 [file 103_2025_4045_MOESM1_ESM.pdf]

Zusätzliches Onlinematerial

Psychische Gesundheit von Kindern und Jugendlichen in Zeiten globaler Krisen:
Ergebnisse der COPSY-Längsschnittstudie von 2020 bis 2024

Abbildung S1.
Längsschnittliche Verlaufsergebnisse des Anteils der Kinder und Jugendlichen mit einer geminderten gesundheitsbezogenen Lebensqualität stratifiziert nach Altersgruppen

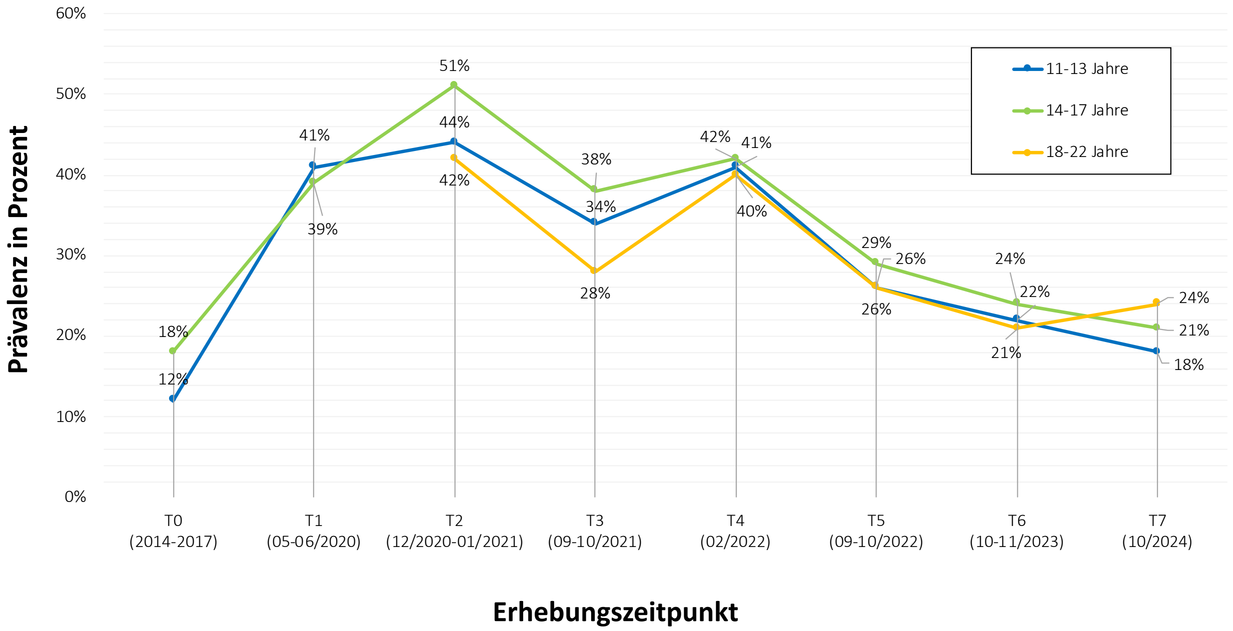

Anmerkung. Eigene Abbildung. Angaben zu 18- bis 22-Jährigen aufgrund der Alterung des Samples erst ab T2 möglich; Statistische Signifikanztestung der Unterschiede: T0 p=0,004; T1 p=0,540; T2 p=0,088; T3 p=0,040; T4 p=0,907; T5 p=0,549; T6 p=0,605; T7 p=0,206.

Abbildung S2.

Längsschnittliche Verlaufsergebnisse des Anteils der Kinder und Jugendlichen mit psychischen Auffälligkeiten stratifiziert nach Altersgruppen

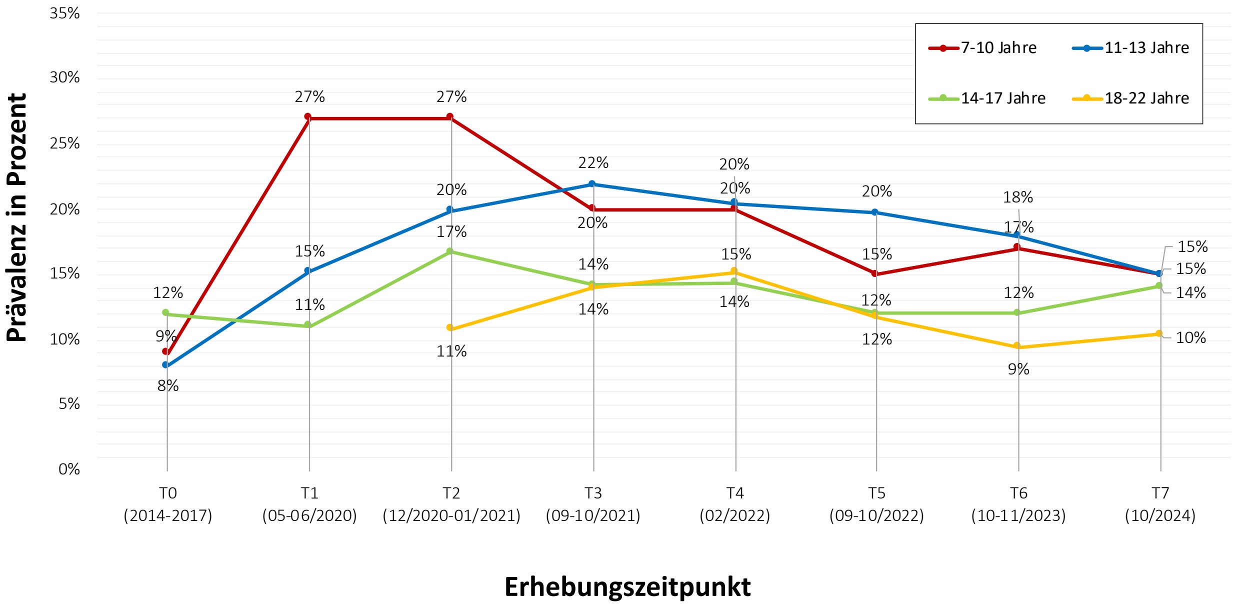

Anmerkung. Eigene Abbildung. Angaben zu 18- bis 22-Jährigen aufgrund der Alterung des Samples erst ab T2 möglich; Statistische Signifikanztestung der Unterschiede: T0 p=0,163; T1 p<0,001; T2 p<0,001; T3 p=0,003; T4 p=0,022; T5 p=0,005; T6 p=0,002; T7 p=0,243.

Abbildung S3.

Längsschnittliche Verlaufsergebnisse des Anteils der Kinder und Jugendlichen mit Angstsymptomen stratifiziert nach Altersgruppen

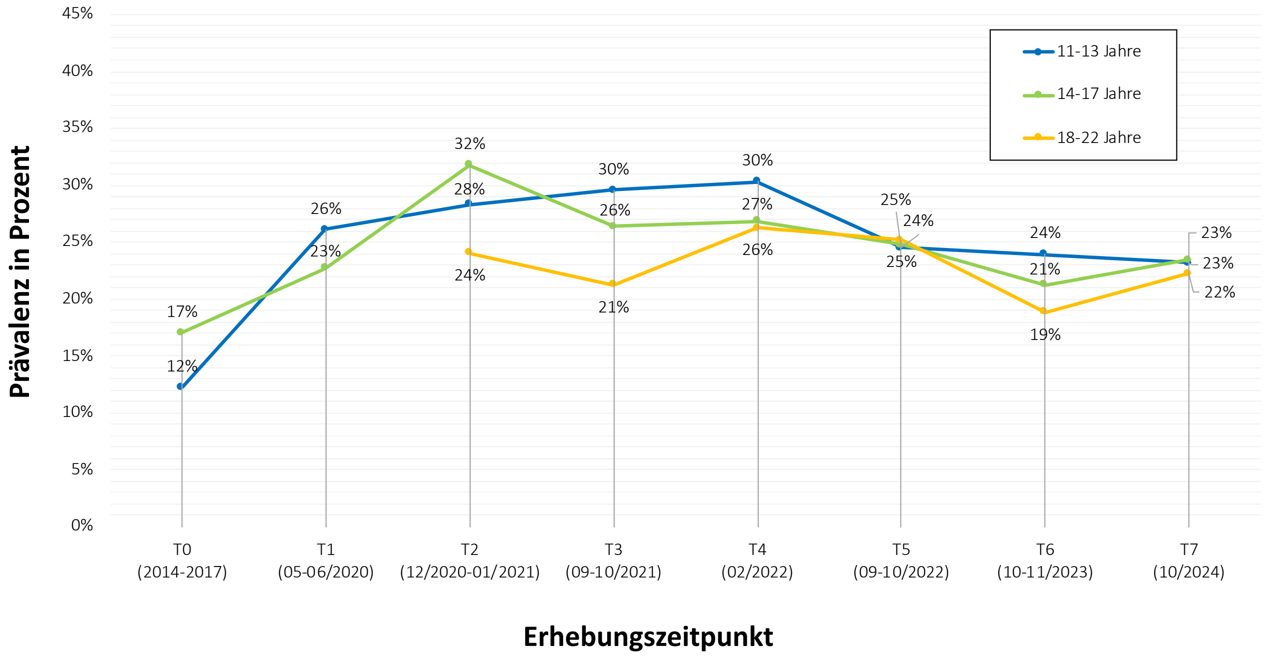

Anmerkung. Eigene Abbildung. Angaben zu 18- bis 22-Jährigen aufgrund der Alterung des Samples erst ab T2 möglich; Statistische Signifikanztestung der Unterschiede: T0 p=0,015; T1 p=0,213; T2 p=0,274; T3 p=0,117; T4 p=0,435; T5 p=0,981; T6 p=0,257; T7 p=0,928.

Abbildung S4.

Längsschnittliche Verlaufsergebnisse des Anteils der Kinder und Jugendlichen mit depressiven Symptomen stratifiziert nach Altersgruppen

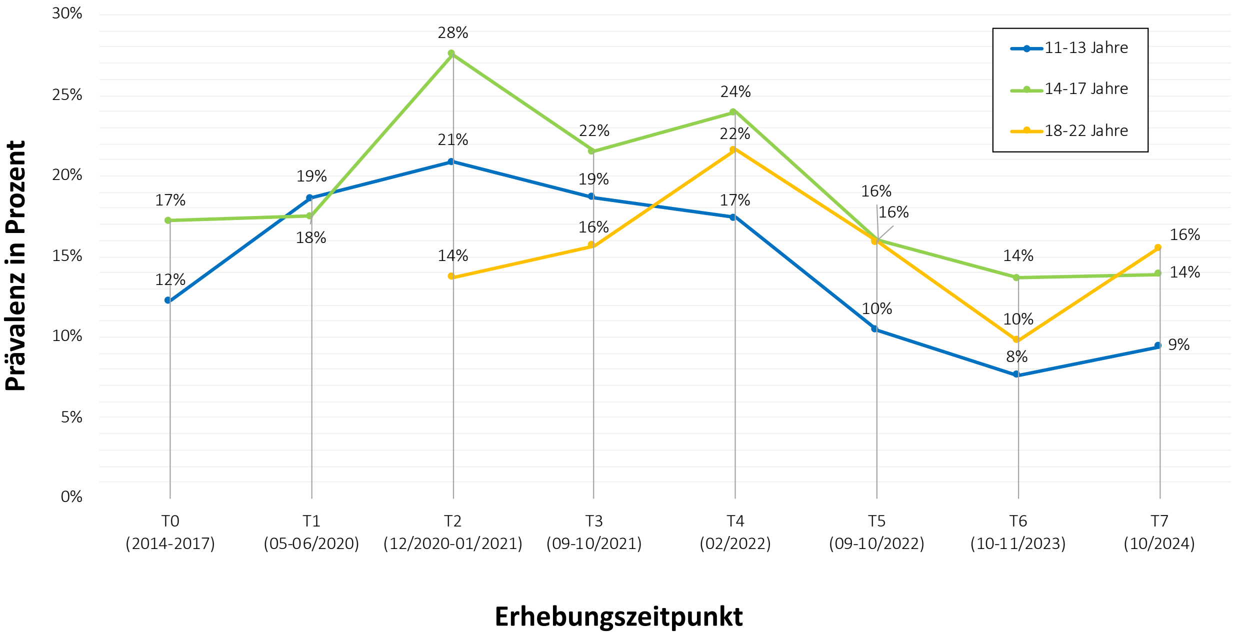

Anmerkung. Eigene Abbildung. Angaben zu 18- bis 22-Jährigen aufgrund der Alterung des Samples erst ab T2 möglich; Statistische Signifikanztestung der Unterschiede: T0 p=0,089; T1 p=0,648; T2 p=0,007; T3 p=0,199; T4 p=0,066; T5 p=0,045; T6 p=0,017; T7 p=0,063.

Abbildung S5.

Längsschnittliche Verlaufsergebnisse des Anteils der Kinder und Jugendlichen mit einer geminderten gesundheitsbezogenen Lebensqualität, psychischen Auffälligkeiten, Angstsymptomen und depressiven Symptomen stratifiziert nach Geschlecht

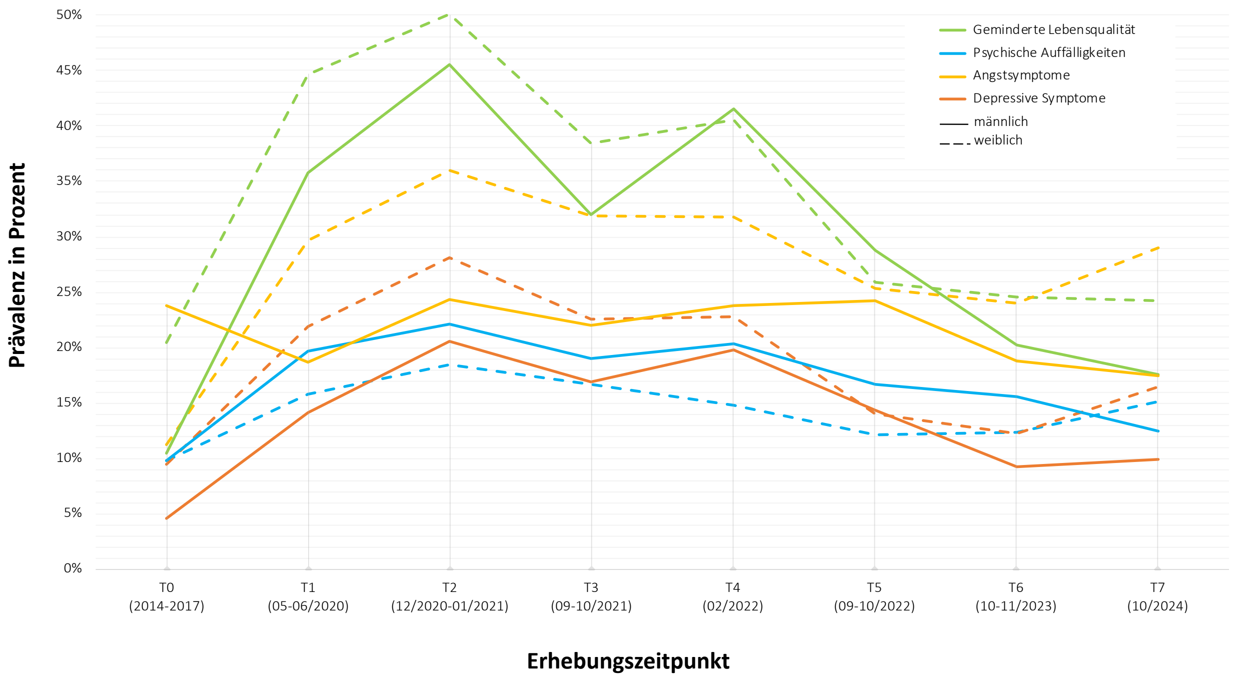

Anmerkung. Eigene Abbildung. Statistische Signifikanztestung der Unterschiede: gLQ: T0 p<0,001; T1 p=0,004; T2 p=0,142; T3 p=0,021; T4 p=0,726; T5 p=0,287; T6 p=0,074; T7 p=0,008; Psychische Auffälligkeiten: T0 p=0,427; T1 p=0,043; T2 p=0,069; T3 p=0,226; T4 p=0,003; T5 p=0,008; T6 p=0,058; T7 p=0,145; Angstsymptome: T0 p<0,001; T1 p<0,001; T2 p<0,001; T3 p<0,001; T4 p=0,003; T5 p=0,673; T6 p=0,030; T7 p<0,001; Depressive Symptome: T0 p<0,001; T1 p=0,001; T2 p=0,004; T3 p=0,014; T4 p=0,218; T5 p=0,837; T6 p=0,087; T7 p=0,002.
